# Mentorship Training is Essential to Advancing Global Health Research

**DOI:** 10.4269/ajtmh.18-0694

**Published:** 2018-11-14

**Authors:** Flora Katz, Roger I. Glass

**Affiliations:** Fogarty International Center, National Institutes of Health, Bethesda, Maryland

We believe well-trained scientists are the key to solving the most difficult global health challenges, ensuring health security from disease threats and improving access to care for those in low- and middle-income countries (LMICs). Today’s complex problems require multidisciplinary approaches and team science, with investigators who are equipped with sophisticated data analysis skills, expertise in ethical research, and other advanced capabilities.

All of this requires effective research training programs that include hands-on field experience with high-quality mentoring. Great mentors are not born. Mentoring skills, like any other, must be developed. This supplement—inspired by a series of mentorship training workshops hosted in LMICs by the faculty of our Fogarty Scholars and Fellows program—includes a mentoring tool kit and details valuable lessons learned about how to foster effective mentors.

We hope this supplement will act as a catalyst for LMIC research organizations to institutionalize mentorship training and to use this publication as a guide to developing successful programs.

What does great mentorship entail? And how can successful mentors be developed? As the Fogarty program’s principal investigators, alumni, and others have laid out in this collection of articles, these are complicated questions whose answers vary by circumstance and location. Working in LMICs poses some unique issues and challenges. But, as the authors suggest, there are some consistent requirements for success.

First, an institution must provide protected time for its staff to devote to preparing for and providing mentorship to its junior members. It should recognize the importance of mentorship by providing mentorship training opportunities and making it part of faculty evaluations, so mentors receive credit for their efforts. Second, the mentor should encourage mentees to question the status quo, propose innovative approaches, and engage in scientific debates with more senior scientists, something that is not always part of the academic culture in LMIC institutions. This will allow for bidirectional learning, which benefits both parties and may inspire novel research approaches. Third, the mentees should be given an ethical framework to foster research integrity and the opportunity to learn by doing, to direct their own research, and to experience their own successes and failures. But a good mentor knows when to step in to provide guidance that can prevent frustration that can lead to burnout. Finally, mentors should cultivate writing skills among mentees so they can publish their results and generate fundable research proposals. In some low-resource settings, there is a shortage of qualified mentors. In those circumstances, peer mentoring can be a viable solution.

Since the 1980s, Fogarty has developed innovative programs to build the next generation of global health scientists in the United States and in LMICs. All have been focused on building research capacity through mentored training, often linked to National Institutes of Health (NIH) research grants. In 2004, we created an initiative to provide American doctoral students in the health professions with a year of mentored research at top-ranked sites in LMICs that have active NIH grants and a proven track record of scientific productivity. Activities include laboratory and clinical studies, as well as epidemiologic and behavioral studies, operations research, and health outcomes evaluations.

Our rationale was that by cultivating an interest in global health research early in participants’ careers, we would encourage them to continue on this path. In 2008, we expanded the program to include postdocs, and in 2010, established a partnership with the Fulbright program to increase fellowship opportunities. Since the program’s inception, we have supported more than 1,000 participants in at least 30 countries, who have published more than 1,200 peer-reviewed publications in a wide variety of infectious and noncommunicable disease areas.^1^ Fellows have come from all branches of medicine and public health, and include infectious disease researchers, veterinarians, engineers, cardiologists, nephrologists, and oncologists, among others; this year, we have our first architect and first lawyer. Originally supported by a single private donation, the program now receives funding from 13 NIH institutes, centers, and offices. There is broad recognition of the importance of providing support to bridge the gap between doctoral work and a solid career pathway in global health research.

Fogarty Fellows report that their successes are largely because of the quality of mentorship they receive during the program. A number of them have noted that the entrée their mentor provided into global networks of scientists who specialize in their research topic has altered the trajectory of their career and enabled them to form long-term partnerships that have boosted their productivity.^2^ Fogarty encourages incorporating mentorship training throughout our programs.

In recognition of the importance of this area, in 2014, Fogarty offered the first Clayton–Dedonder Mentorship Fellowships as a supplement opportunity to the Global Fellows and Scholars Program, using funds the World AIDS Foundation provided to the NIH. The program’s goal is to enhance mentorship training for outstanding former Fellows working in the human immunodeficiency virus (HIV)/AIDs area who are now supervising other students. In 2017, we made this supplement available to all of our HIV/AIDs-relevant programs to develop mentorship and leadership courses at their institutions.^3^

One positive outcome of this research training has been that our fellows and grantees have excelled at becoming leaders in research and biomedical science, and assumed visible positions of responsibility as deans, professors, chancellors, and ministers of health, science, and technology. Some of these fellow alumni and grantees have led programs and coauthored articles included in this special issue on mentoring in LMICs. By becoming mentors and leaders, they are increasing their impact to improve health while advancing research and innovation. It is our hope that this collection of articles will provide a stimulus for increased funding to fill this critical need, and that it will be a useful guide for development of robust mentorship training programs at LMIC institutions.
